# Silicon‐Enhanced Adipogenesis and Angiogenesis for Vascularized Adipose Tissue Engineering

**DOI:** 10.1002/advs.201800776

**Published:** 2018-09-30

**Authors:** Xiaoya Wang, Long Gao, Yan Han, Min Xing, Cancan Zhao, Jinliang Peng, Jiang Chang

**Affiliations:** ^1^ State Key Laboratory of High Performance Ceramics and Superfine Microstructure Shanghai Institute of Ceramics Chinese Academy of Sciences 1295 Dingxi Road Shanghai 200050 P. R. China; ^2^ University of Chinese Academy of Sciences 19 Yuquan Road Beijing 100049 P. R. China; ^3^ School of Pharmacy Shanghai Jiao Tong University 800 Dongchuan Road Shanghai 200240 P. R. China

**Keywords:** adipogenesis, adipogenic differentiation, adipose tissue engineering, angiogenesis, dedifferentiation

## Abstract

The enhancement of adipogenic differentiation of bone marrow mesenchymal stem cells (BMSCs) and sufficient vascularization remain great challenges for the successful reconstruction of engineered adipose tissue. Here, the bioactive effects of silicon (Si) ions on adipogenic differentiation of human BMSCs (HBMSCs) and the stimulation of vascularization during adipose tissue regeneration are reported. The results show that Si ions can enhance adipogenic differentiation of HBMSCs through the stimulation of the expression of adipogenic differentiation switches such as peroxisome proliferator‐activated receptor γ and CCAAT/enhancer‐binding protein α. Furthermore, Si ions can enhance both angiogenesis and adipogenesis, and inhibit dedifferentiation of cocultured adipocytes by regulating the interactions between HBMSC‐derived adipocytes and human umbilical vein endothelial cells, in which the promotion of the expression of insulin‐like growth factor 1 and vascular endothelial growth factor plays vital roles. The in vivo studies further demonstrate that the designed composite hydrogel with the ability to release bioactive Si ions clearly stimulates neovascularization and adipose tissue regeneration. The study suggests that Si ions released from biomaterials are important chemical cues for adipogenic differentiation and biomaterials with the ability to release Si ions can be designed for adipose tissue engineering.

## 1 Introduction

Adipose tissue engineering strategies have shown great potential in the treatment of soft tissue defects originated from severe burns, trauma, tumor resection, and congenital deformities.^1^ Bone marrow mesenchymal stem cells (BMSCs) are a promising cell type for tissue engineering due to their strong proliferation ability and multiple differentiation potentials, which are able to differentiate into different cell linages such as osteoblasts, adipocytes, chondrocytes under appropriate stimuli conditions.^2^ Pittenger et al. first reported that BMSCs can differentiate into adipogenic linage by treatment with l‐methyl‐3‐isobutylxanthine (IBMX), dexamethasone, insulin, and indomethacin, which is considered as the universal method for adipogenic induction.[[qv: 2b]] In recent years, some improved methods have been developed to accelerate adipogenic differentiation of MSCs.[[qv: 1c,3]] However, many studies have shown that reconstruction of adipose tissue generated by adipogenic‐differentiated MSCs is limited and the amount of newly formed adipose tissue was insufficient to meet the requirement of adipose tissue reconstruction.[[qv: 1a,4]] One problem may be the low adipogenic differentiation efficiency of MSCs, and gradual decrease of adipogenic differentiation capability of MSCs during extensive in vitro expansion and long‐term inductive differentiation by using the conventional chemical induction, which usually takes 2–3 weeks for MSCs to differentiate into adipocytes.[[qv: 3a,5]] Therefore, how to increase the efficiency of adipogenic differentiation of MSCs is of the utmost importance.[[qv: 1c,3b,d]] In addition to the insufficient adipogenic differentiation potential of MSCs, it has been widely recognized that the lack of sufficient vascularization is also one of the key problems resulting in failure of long‐term survival of regenerated adipose‐like tissue.[[qv: 1a,6]] Native fatty tissue is highly vascularized and requires rich vascular supply to support its highly metabolic activity.[[qv: 1b,c]] Therefore, for the tissue engineering scaffolds whose size is larger than the physiological diffusion limit of oxygen and nutrients, the newly formed adipose tissue is at risk to suffer from reabsorption and necrosis due to the lack of vascular supply.[[qv: 1c,7]] In order to solve the problem of vascularization, different methods have been investigated. A tissue engineering chamber (TEC) model has been reported, in which vascularized adipose tissue was generated by implanting a hollow chamber containing an adipose tissue flap subcutaneously in the groin of the rat.^8^ Based on the animal study, a clinical study has been conducted, which confirmed the effectiveness of the application of the TEC in human, but limitation still exists, including complicated surgical procedure and low success rate.^9^ Furthermore, considering the coexistence of adipocytes and endothelial cells in native fatty tissues, previous studies have demonstrated that the coculture of MSC‐derived adipocytes and endothelial cells might be an effective strategy to reconstruct engineered adipose tissue with better vascularization.[[qv: 1c]] However, the problem with the coculture strategy is that the differentiated adipocytes often undergo rapid dedifferentiation during in vitro coculture.[[qv: 1c,10]] This is also an important issue that affect adipose tissue construction, and up to now, there seems no more effective strategies inhibiting the dedifferentiation of differentiated adipocytes.[[qv: 1b]]

In recent years, more and more evidences show that bioactive ions play important roles in regulating cell behaviors including cell differentiation.^11^ Some studies have shown that zinc promoted adipogenesis of rat adipocytes and stimulated the conversion of glucose to lipids in 3T3‐L1 fibroblasts and adipocytes in vitro and in vivo,^12^ suggesting that some bioactive ions indeed can function as stimuli in promoting adipogenic differentiation. It is known that silicon (Si) as an essential trace element participates in bone and skin tissue regeneration.[[qv: 11a,13]] Many studies have demonstrated that Si ions have the activity to enhance osteogenic differentiation of human BMSCs (HBMSCs).[[qv: 13a]] In addition, it has been reported that Si ions can stimulate angiogenesis of endothelial cells, and silicate‐based bioactive materials can enhance wound healing by regulating cell–cell interactions, stem cell migration and stimulating blood vessel formation.[[qv: 13b,d,14]] These results suggest that Si ions may play a multifunctional role by enhancing stem cell differentiation, activating cell–cell interactions and stimulating angiogenesis. Based on the consideration of the multiple roles of Si ions in tissue regeneration, we hypothesize that 1) Si ions may enhance adipogenic differentiation of HBMSCs, thereby accelerating adipose tissue regeneration; 2) Si ions may enhance adipose tissue regeneration by stimulating angiogenesis; 3) Si ions may regulate the interactions between HBMSC‐derived adipocytes and endothelial cells and inhibit the dedifferentiation of cocultured adipocytes, and therefore promote both adipogenesis and angiogenesis.

To confirm our hypotheses, in this study, we first investigated the bioactive effect of Si ions on adipogenic differentiation of HBMSCs, followed by exploring the effect of Si ions on the interactions of HBMSC‐derived adipocytes and human umbilical vein endothelial cells (HUVECs), and specifically synergistic effect of Si ions on enhancing angiogenesis of cocultured HUVECs and inhibiting the dedifferentiation of cocultured adipocytes. Finally, based on the bioactive concentration of Si ions identified, we reconstructed an engineered adipose tissue in a nude mice subcutaneous implant model by combining the cocultured HBMSC‐derived adipocytes and HUVECs in calcium silicate/alginate composite hydrogel with the ability to release bioactive Si ions.

## 2 Results

### 2.1 Effects of Si Ions on Cell Proliferation and Morphology of HBMSCs and HUVECs

**Figure 1:**
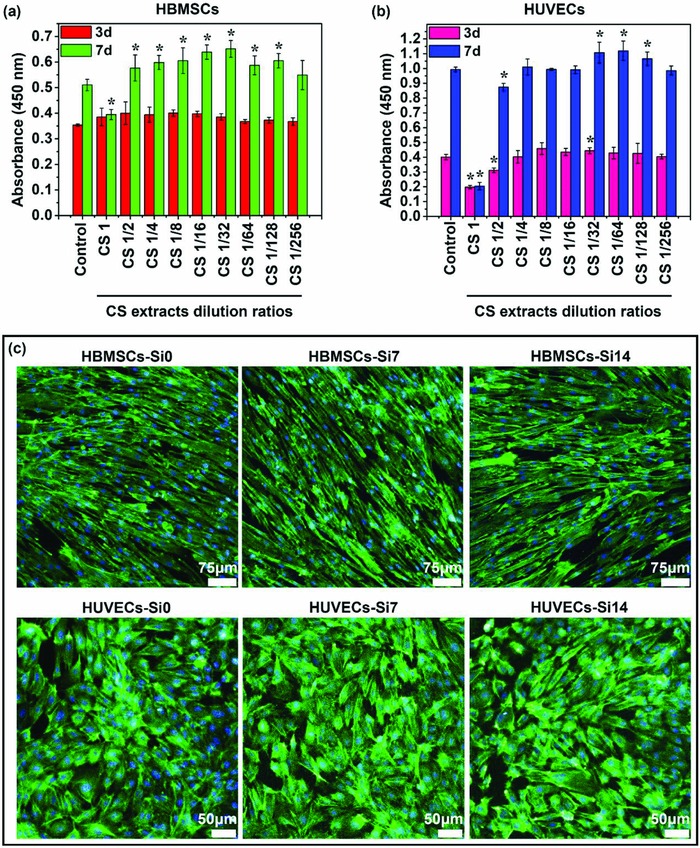
Cell proliferation and morphology of HBMSCs and HUVECs cultured with different concentrations of Si ions. a,b) CCK‐8 assay indicated that Si ions in certain concentration range can stimulate the proliferation of HBMSCs and HUVECs (*n* = 6 for both the groups; *p < 0.05). c) Actin cytoskeleton staining assay showed that both concentrations of Si ions (7 and 14 µg mL^−1^) did not affect the morphology of HBMSCs and HUVECs. Scale bar, 75 µm for HBMSCs and 50 µm for HUVECs. The actin cytoskeletons were stained with FITC–Phalloidin (green), and nucleus was counterstained by using DAPI (blue).

The effects of ions released from calcium silicate (CS) on cell proliferation were evaluated and the results are shown in **Figure** **1**a,b. It is clear to see that at dilution ratios from 1/4 to 1/256 (Si‐ion concentration: 0.5–29.27 µg mL^−1^), CS extracts showed no cytotoxicity for both HBMSCs and HUVECs, but CS extracts without dilution (CS1) showed a certain degree of cytotoxicity for both type of the cells. More interestingly, CS extracts at a certain dilution range revealed a stimulatory effect on cell proliferation, whereas the active concentrations for the stimulation were different for different cell types (Figure 1a,b). The CS extracts diluted from 1/2 to 1/128 (Si‐ion concentration: 0.95–59.57 µg mL^−1^) significantly stimulated HBMSC proliferation on day 7 with 13–28% increase as compared to the control group (Figure 1a). For HUVECs, CS extracts at dilution ratios from 1/32 to 1/128 (Si‐ion concentration: 0.95–3.67 µg mL^−1^) stimulated cell proliferation by 7–13% as compared to the control group. At dilution ratios of 1/4 to 1/16 (Si‐ion concentration: 7.36–29.27 µg mL^−1^), CS extracts maintained the viability of HUVECs which indicated that these concentrations were not cytotoxic to HUVECs (Figure 1b). In order to determine the bioactive concentrations of the Si ions for the stimulation of cell proliferation, CS extracts diluted with Dulbecco's modified Eagle medium (DMEM) by a series of gradient dilution at the ratios from 1 to 1/256 were measured and the results were listed in **Table** **1**. It can be seen that Si concentrations in all the dilutions of CS extracts were much higher than those in the control medium (DMEM). In contrast, the Ca and P ions' concentrations of most dilutions were at similar level as that in the control medium except the original extract and 1/2 and 1/4 dilutions, which were lower than the control medium. These results suggest that at the dilution range from 1/8 to 1/256, Si ions play a key role in regulating cell proliferation, and the bioactive Si‐ion concentration for the stimulation of HBMSC proliferation is in the range between 0.95 and 59.57 µg mL^−1^, and that for the stimulation of HUVEC proliferation is in the range of 0.95–3.67 µg mL^−1^, which is much narrow than that for HBMSCs. The fluorescence images of actin cytoskeleton staining revealed the morphology of HBMSCs and HUVECs cultured with different concentrations of Si ions (7 and 14 µg mL^−1^) for 7 days (Figure 1c). It can be seen that HBMSCs demonstrated an elongated, fibroblastic appearance, and HUVECs showed a typical cobblestone‐like appearance, indicating that Si ions did not affect the morphology of HBMSCs and HUVECs (Figure 1c).

**Table 1:** Ion concentration of calcium silicate extracts diluted at ratios from 1 to 1/256 using DMEM medium

	Si [µg mL^−1^]	Ca [µg mL^−1^]	P [µg mL^−1^]
DMEM	0.02 ± 0.00	58.15 ± 0.60	25.86 ± 0.32
CS1	120.52 ± 0.43^a)^	5.97 ± 0.77	5.96 ± 0.26
CS1/2	59.57 ± 0.15a)	33.09 ± 0.55	15.44 ± 0.32
CS1/4	29.27 ± 0.53a)	45.76 ± 0.42	20.75 ± 0.02
CS1/8	14.53 ± 0.21a)	56.54 ± 0.89	24.54 ± 0.30
CS1/16	7.36 ± 0.13a)	57.82 ± 0.24	25.20 ± 0.21
CS1/32	3.67 ± 0.23a)	58.01 ± 0.05	25.15 ± 0.25
CS1/64	1.90 ± 0.19a)	58.17 ± 0.33	25.85 ± 0.10
CS1/128	0.95 ± 0.10a)	58.30 ± 0.55	25.97 ± 0.15
CS1/256	0.50 ± 0.03a)	58.55 ± 0.81	26.11 ± 0.07

^a)^ Indicates that the Si‐ion concentrations in CS extracts were significantly higher than those in DMEM (*p* < 0.05).

### 2.2 Effects of Si Ions on Adipogenic Differentiation of HBMSCs

**Figure 2:**
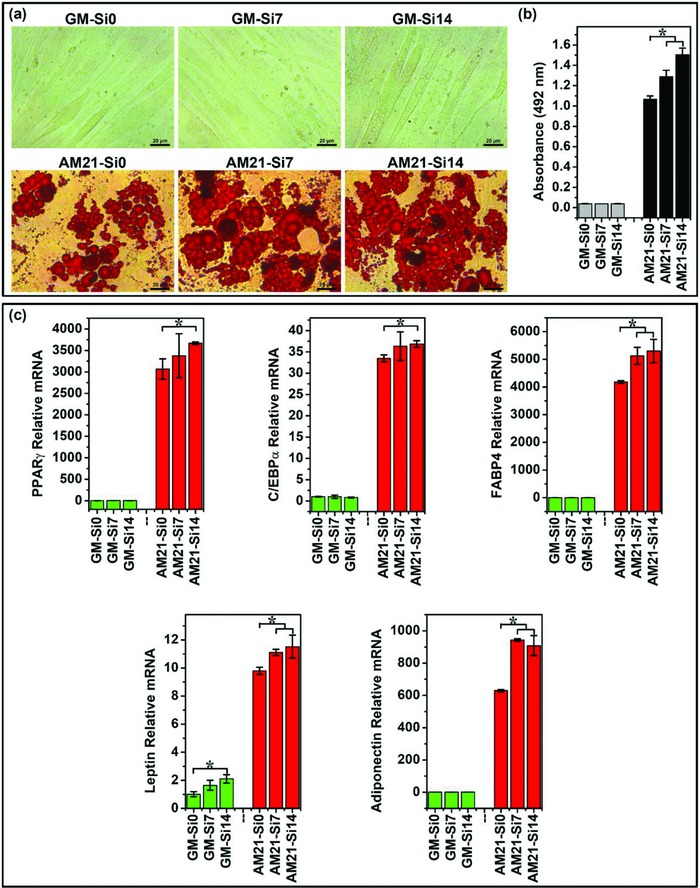
Oil Red O staining and adipogenic gene expression of HBMSCs cultured in different mediums with or without Si ions. a) Oil Red O staining of cells showed that Si ions stimulated the increase of lipid accumulation. Lipid droplets were stained in red. Scale bar, 20 µm. b) Quantitative analysis of Oil Red O staining showed that Si ions significantly stimulated lipid accumulation (*n* = 3 for both the groups; *p < 0.05). c) Quantitative PCR analysis showed that Si ions enhanced the adipogenic marker gene of PPARγ, C/EBPα, FABP4, leptin, and adiponectin expression of HBMSCs (*n* = 3 for both the groups; *p < 0.05). GM‐Si0, GM‐Si7, GM‐Si14: HBMSCs cultured with growth medium containing different concentrations of Si ions (0, 7, and 14 µg mL^−1^) without differentiation inducer for 21 days, respectively; AM21‐Si0, AM21‐Si7, AM21‐Si14: HBMSCs cultured with adipogenic differentiation medium containing different concentrations of Si ions (0, 7, and 14 µg mL^−1^), respectively, for 21 days.

The effect of Si ions on adipogenic differentiation of HBMSCs was analyzed and the results are shown in **Figure** **2**. The Oil Red O staining showed that when HBMSCs were cultured with adipogenic differentiation medium in presence of Si ions for 21 days, the adipogenic differentiation of HBMSCs was clearly enhanced as compared with cells only cultured in adipogenic medium without Si ions (Figure 2a,b). As shown in Figure 2b, the lipid accumulation in cells treated with media containing 7 and 14 µg mL^−1^ Si ions (AM21‐Si7, AM21‐Si14) was increased by 21% and 41%, respectively, as compared to that in the cells cultured without Si ions (AM21‐Si0). The results suggest that Si ions have significant contribution to adipogenic differentiation, although their relative effect was smaller than the effect of adipogenic medium. Under normal growth condition without adipogenic differentiation inducer, Si ions could not stimulate lipid accumulation of HBMSCs (Figure 2a,b GM group). To further confirm whether Si ions could enhance adipogenic gene expression during differentiation, two transcriptional factors peroxisome proliferator‐activated receptor γ (PPARγ) and CCAAT/enhancer‐binding protein α (C/EBPα), which play key roles in driving adipogenic differentiation,^15^ and several markers of terminal adipogenic differentiation including fatty acid binding protein 4 (FABP4), leptin, and adiponectin were detected by quantitative real‐time polymerase chain reaction (qRT‐PCR) (Figure 2c). Similar to the result of Oil Red O staining, Si ions were observed to significantly upregulate the gene expression of PPARγ, C/EBPα, FABP4, leptin, and adiponectin in adipogenic medium, which indicated that Si ions do promote adipogenic differentiation of HBMSCs (Figure 2c). In contrast, in normal growth medium, no upregulation of adipogenic gene expression was observed both with and without Si ions, suggesting that Si ions are not an inducer, and rather an enhancer of the adipogenic differentiation of HBMSCs.

**Figure 3:**
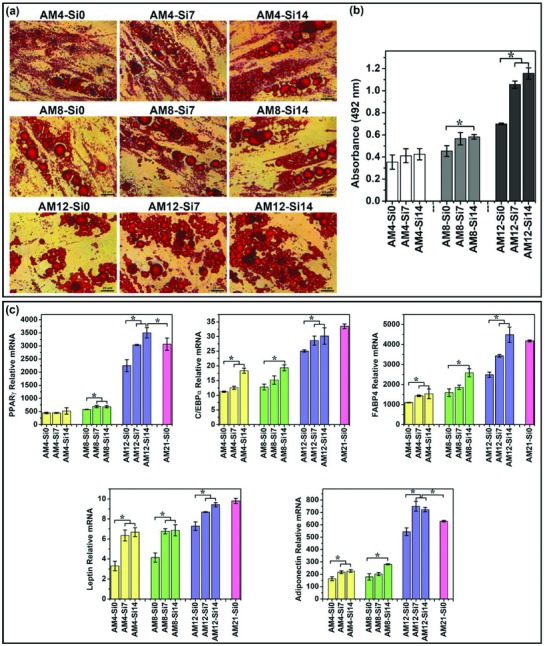
The effects of Si ion as a differentiation enhancer on the stimulation of adipogenic differentiation. a) Oil Red O staining of cells showed that Si ions stimulated the increase of lipid accumulation. Lipid droplets were stained in red. Scale bar, 20 µm. b) Quantitative analysis of Oil Red O staining showed that Si ions significantly stimulated lipid accumulation (*n* = 3 for both the groups; *p < 0.05). c) Quantitative PCR analysis showed that Si ions enhanced the adipogenic marker gene of PPARγ, C/EBPα, FABP4, leptin, and adiponectin expression of HBMSCs (*n* = 3 for both the groups; *p < 0.05). AM4‐Si7, AM4‐Si14: HBMSCs cultured with adipogenic differentiation medium alone for 4 days, and then further cultured up to 21 days in growth medium containing different concentrations of Si ions (0, 7, and 14 µg mL^−1^), respectively; AM8‐Si0, AM8‐Si7, AM8‐Si14: HBMSCs cultured with adipogenic differentiation medium alone for 8 days, and then further cultured up to 21 days in growth medium containing different concentrations of Si ions (0, 7, and 14 µg mL^−1^), respectively; AM12‐Si0, AM12‐Si7, AM12‐Si14: HBMSCs cultured with adipogenic differentiation medium alone for 12 days, and then further cultured up to 21 days in growth medium containing different concentrations of Si ions (0, 7, and 14 µg mL^−1^), respectively; AM21‐Si0: HBMSCs cultured in adipogenic differentiation medium without Si ions for 21 days.

In order to further explore the effects of Si ion as a differentiation enhancer on the stimulation of adipogenesis, HBMSCs were first cultured with adipogenic differentiation medium alone for 4, 8, and 12 days, respectively, and then further cultured up to 21 days in normal growth medium containing different concentrations of Si ions (7 and 14 µg mL^−1^). The results showed that when cells have been induced to adipogenic differentiation for certain time, then even without differentiation inducer, Si ions could also enhance lipid accumulation and expression of adipogenic genes of HBMSCs (**Figure** **3**). This result indicates that once the cells are pushed into the direction of adipogenic differentiation, Si ions can push the cells to continue adipogenic differentiation even without the differentiation inducer. More interestingly, after treating cells with adipogenic differentiation medium (AM) alone for 12 days, then replacing AM with growth medium containing Si ions and culturing for up to 21 days, the degree of adipogenic differentiation enhanced by Si ions could reach or even exceed the differentiation level of the cells cultured in adipogenic medium without Si ions for 21 days (Figure 3c, AM12‐Si7, AM12‐Si14, AM21‐Si0).

### 2.3 Effects of Si on Angiogenesis of Cocultured HBMSC‐Derived Adipocytes and HUVECs

**Figure 4:**
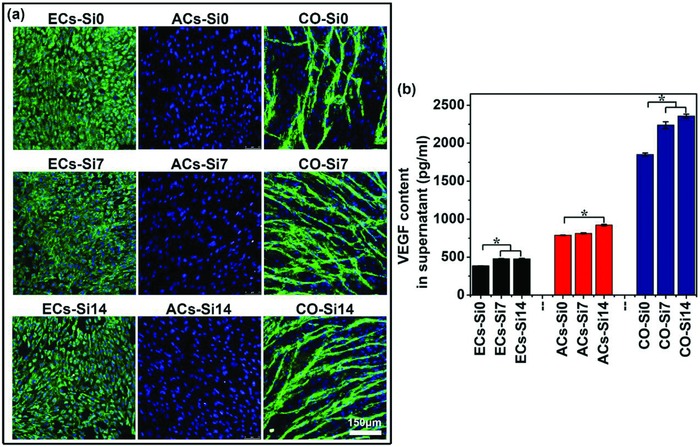
Immunofluorescence staining of vWF and VEGF secretion of monocultured cells and cocultured cells treated with or without Si ions. a) vWF staining showed that clear capillary‐like networks were formed in the cocultured cells but not in the monocultured HUVECs and adipocytes. In addition, Si ions stimulated the network formation. vWF was stained in green and nuclei in blue with DAPI. Scale bar, 150 µm. b) Quantitative ELISA analysis showed that Si ions enhanced the VEGF secretion of monocultured cells and cocultured cells in culture medium (*n* = 3 for both the groups; *p < 0.05). ECs‐Si0, ECs‐Si7, ECs‐Si14: HUVECs (ECs) cultured with growth medium containing different concentrations of Si ions (0, 7, and 14 µg mL^−1^) for 3 days; ACs‐Si0, ACs‐Si7, ACs‐Si14: HBMSCs cultured in adipogenic differentiation medium with different concentrations of Si ions (0, 7, and 14 µg mL^−1^) for 15 days for the development of the adipocytes' (ACs) phenotype; CO‐Si0, CO‐Si7, CO‐Si14: HBMSCs first cultured in adipogenic differentiation medium with different concentrations of Si ions (0, 7, and 14 µg mL^−1^) for 12 days for adipogenic differentiation, and then cocultured with HUVECs containing different concentrations of Si ions (0, 7, and 14 µg mL^−1^) for 3 days.

To explore the effects of Si ions on cell–cell interactions and angiogenesis, an in vitro coculture model with HBMSC‐derived adipocytes and HUVECs was applied. von Willebrand factor (vWF) immunostaining showed clear capillary‐like networks formation in cocultured cells, while there was no clear network formed in monocultured adipocytes or HUVECs. Furthermore, the addition of Si ions in the coculture medium remarkably stimulated the capillary‐like network formation, which indicated that Si ions enhanced angiogenesis of the cocultured cells (**Figure** **4**a). Enzyme‐linked immunosorbent assay (ELISA) analysis also showed that although Si ions also promoted vascular endothelial growth factor (VEGF) secretion of monocultured adipocytes and HUVECs as compared to cells cultured without Si ions, a significantly higher increase of the VEGF secretion was observed in cocultured cells in the presence of Si ions as compared to monocultured cells (Figure 4b). This result indicates a possible activation of the interactions of the cocultured adipocytes and HUVECs by Si ions which contribute to angiogenesis.

**Figure 5:**
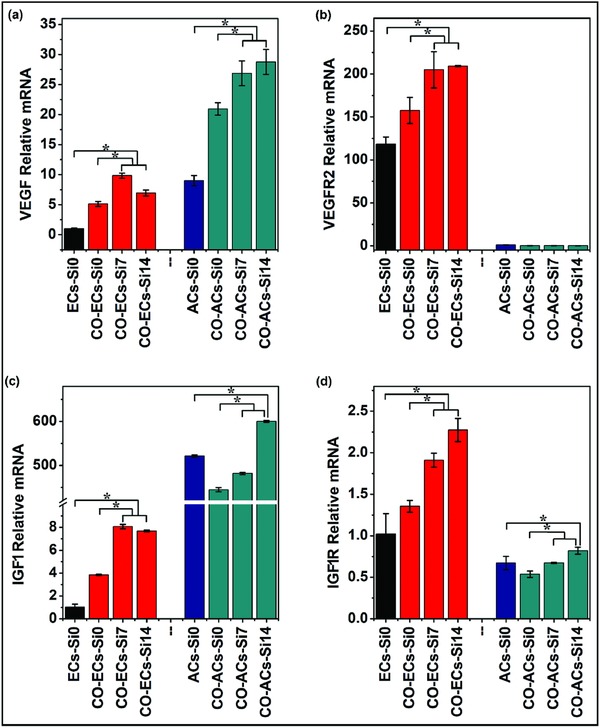
Angiogenic and adipogenic gene expression of monocultured cells and cocultured cells treated with or without Si ions. HBMSCs were first cultured in adipogenic differentiation medium for 12 days for the development of the adipocyte phenotype, and then cocultured with HUVECs containing different concentrations of Si ions (0, 7, and 14 µg mL^−1^) for 3 days. a–d) Quantitative PCR analysis showed that Si ions stimulated the gene expression of VEGF, VEGFR2, IGF1, and IGF1R of cocultured HUVECs and cocultured adipocytes (*n* = 3 for both the groups; *p < 0.05). ECs‐Si0: monocultured HUVECs without Si ions; CO‐ECs‐Si0, CO‐ECs‐Si7, CO‐ECs‐Si14: cocultured HUVECs incubated with different concentrations of Si ions (0, 7, and 14 µg mL^−1^); ACs‐Si0: monocultured adipocytes without Si ions; CO‐ACs‐Si0, CO‐ACs‐Si7, CO‐ACs‐Si14: cocultured adipocytes incubated with different concentrations of Si ions (0, 7, and 14 µg mL^−1^).

In order to elucidate possible mechanism of Si ion activated angiogenesis and cell–cell interactions, the two type of cells were separated after coculturing for 3 days and the expression of angiogenic genes such as VEGF and its receptor VEGFR2, insulin‐like growth factor 1 (IGF1) and its receptor IGF1R from the separated cells were measured (**Figure** **5**). Similar as observed in VEGF protein secretion, compared with monocultured HUVECs and adipocytes, co‐HUVECs and co‐adipocytes showed clear upregulation in VEGF expression, respectively (Figure 5a). More interestingly, the VEGF expression in co‐adipocytes was much higher than that in co‐HUVECs in Si containing medium (Figure 5a). In contrast, Si ions only stimulated VEGFR2 expression in co‐HUVECs, and did not stimulate the VEGFR2 expression in co‐adipocytes (Figure 5b).

IGF1, an important growth factor, acts through its receptor IGF1R on the cytomembrane to stimulate endothelial cells migration and regulate angiogenesis.^16^ Therefore, we also analyzed the messenger RNA (mRNA) levels of IGF1 and IGF1R genes (Figure 5c,d). Similar to the result of VEGF and VEGFR2 expression, Si ions strongly upregulated the expression of IGF1 in co‐adipocytes (Figure 5c), and IGF1R expression in co‐HUVECs (Figure 5d).

### 2.4 Effects of Si on the Inhibition of Dedifferentiation of Cocultured Adipocytes

**Figure 6:**
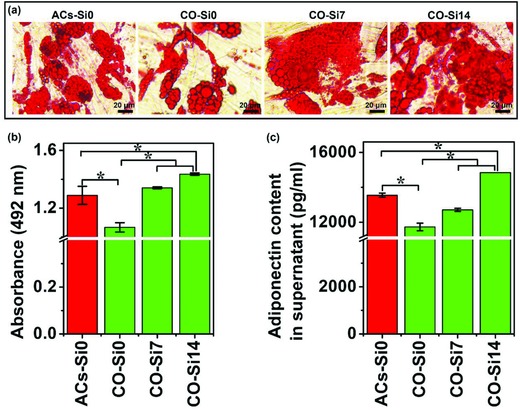
Oil Red O staining and adiponectin secretion of monocultured cells and cocultured cells treated with or without Si ions. a) Oil Red O staining of cells showed that Si ions stimulated the increase of lipid accumulation in the coculture system and inhibited the dedifferentiation of cocultured adipocytes. Lipid droplets were stained in red. Scale bar, 20 µm. b) Quantitative analysis of Oil Red O staining (*n* = 3 for both the groups; *p < 0.05). c) Quantitative ELISA analysis showed that Si ions enhanced the adiponectin secretion of cocultured cells in culture medium (*n* = 3 for both the groups; *p < 0.05). ACs‐Si0: HBMSCs cultured in adipogenic differentiation medium without Si ions for 12 days for the development of the adipocytes' (ACs) phenotype; CO‐Si0, CO‐Si7, CO‐Si14: HBMSCs first cultured in adipogenic differentiation medium with different concentrations of Si ions (0, 7, and 14 µg mL^−1^) for 12 days for adipogenic differentiation, and then cocultured with HUVECs containing different concentrations of Si ions (0, 7, and 14 µg mL^−1^) for 3 days.

To explore the effects of Si ions on the inhibition of dedifferentiation of cocultured adipocytes, we further compared the degree of adipogenic differentiation between the cocultured cells with or without Si ions. Oil Red O staining and ELISA analysis of adiponectin secretion of mono‐ and cocultured cells were shown in **Figure** **6**. It is clear to see that cocultured adipocytes showed reduced Oil Red O staining and adiponectin secretion as compared to monocultured adipocytes, indicating a drastic dedifferentiation of cocultured adipocytes (Figure 6, CO‐Si0). Interestingly, we found that Si ions significantly stimulated lipid accumulation and adiponectin secretion of cocultured adipocytes, and the highest effective Si‐ion concentration for stimulation was 14 µg mL^−1^, which resulted in a remarkable increase of lipid accumulation and adiponectin secretion even higher than that of the monocultured adipocytes in adipogenic medium without Si ions (Figure 6, CO‐Si14). In addition, IGF1, known also as an important adipogenic factor[[qv: 3c,17]] besides the role in angiogenesis, was found clearly downregulated in cocultured adipocytes (ACs) as compared to monocultured adipocytes (Figure 5c, CO‐ACs‐Si0), which also indicates the dedifferentiation of cocultured adipocytes. However, Si ions at the concentration of 14 µg mL^−1^ also significantly stimulated IGF1 expression in cocultured adipocytes as compared to the cocultured cells without Si, which was even higher than that of the monocultured adipocytes in adipogenic medium without Si ions (Figure 5c, CO‐ACs‐Si14). These results suggest that Si ions are able to inhibit the dedifferentiation of cocultured adipocytes by stimulating lipid accumulation and adiponectin secretion, and promoting the expression of the adipogenic factor IGF1.

### 2.5 Effects of Si Ions on In Vivo Adipose Tissue Regeneration

**Figure 7:**
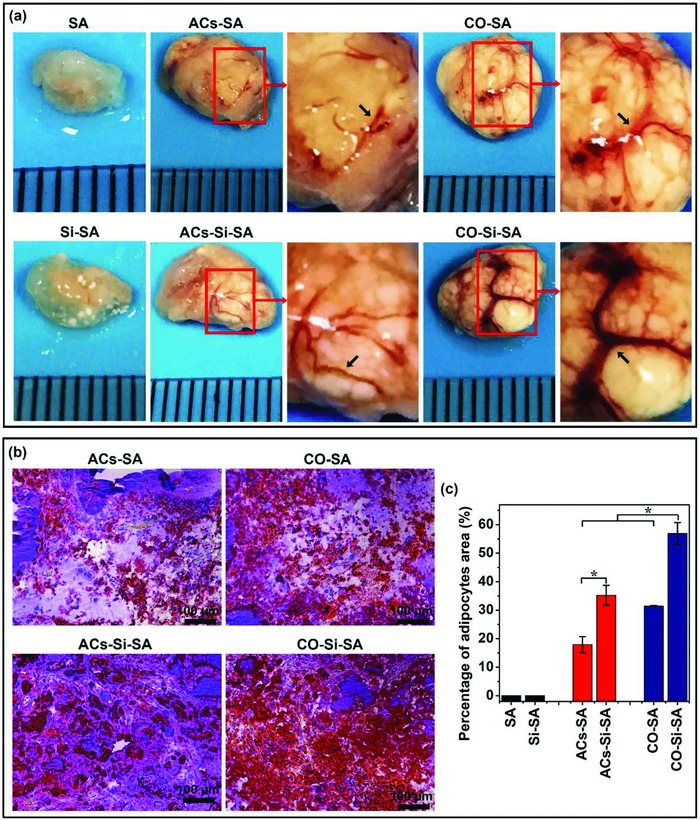
Tissue appearance and Oil Red O staining of engineered adipose tissue in a nude mice subcutaneous implant model by combining the monocultured cells or cocultured cells in calcium silicate/alginate composite hydrogel with the ability to release bioactive Si ions. a) Appearance of engineered adipose tissue showed that Si ions stimulated adipose tissue growth and vascular network (black arrows) formation. High magnification corresponded to boxed area (red) in the low magnification images. b) Oil Red O staining of implants showed that Si ions promoted adipose tissue regeneration in vivo. Lipid droplets were stained in red and nucleus were counterstained in blue with hematoxylin. Scale bar, 100 µm. c) Quantitative analysis of Oil Red O staining of the implants indicated that Si ions stimulated adipose tissue formation (*n* = 5 for both the groups; *p < 0.05). SA: hydrogels without cells; Si‐SA: Si ion–released hydrogels without cells; ACs‐SA: hydrogels with monocultured adipocytes; ACs‐Si‐SA: Si ion–released hydrogels with monocultured adipocytes; CO‐SA: hydrogels with cocultured adipocytes and HUVECs; CO‐Si‐SA: Si ion–released hydrogels with cocultured adipocytes and HUVECs.

In order to further investigate the stimulatory effects of Si ions on vascularized adipose tissue engineering in vivo, we reconstructed an engineered adipose tissue in a nude mice subcutaneous implant model by combining the cocultured HBMSC‐derived adipocytes and HUVECs in calcium silicate/alginate composite hydrogel with the ability to release bioactive Si ions. **Figure** **7**a showed the appearance of engineered adipose tissue after 8 weeks transplantation. It is clear to see that the implanted hydrogel with monocultured adipocytes or cocultured cells all showed obvious formation of adipose‐like tissues as compared with the pure hydrogel implantation, and Si ion–releasing hydrogel with cocultured cells showed a larger and more complete adipose tissue morphology than that with monocultured adipocytes (Figure 7a). Oil red O staining further confirmed the optical observation that Si ions remarkably enhanced the formation of adipose‐like tissue in hydrogels with both mono‐ and cocultured adipocytes, and coculture group showed higher adipose tissue formation than monoculture group (Figure 7b,c).

### 2.6 Effects of Si Ions on In Vivo Neovascularization

**Figure 8:**
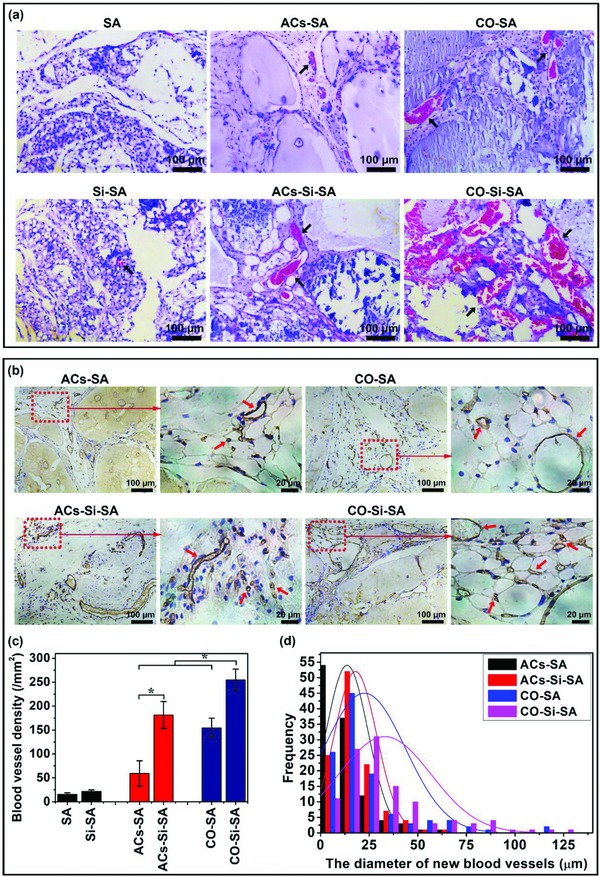
H&E staining and CD31 immunohistochemical staining of engineered adipose tissue implants. a) H&E staining showed that Si ions stimulated neovascularization in vivo. Blood vessels (black arrows) was stained in red and nuclei in blue. Scale bar, 100 µm. b) CD31 immunohistochemical staining showed that Si ions stimulated numerous microvessels' formation. Blood vessels (red arrows) was stained in brown and nuclei in blue. High magnification corresponded to boxed area (red) in the low magnification images. Scale bar, 100 µm for low magnification groups, 20 µm for the high magnification groups. c) Quantitative analysis of blood vessel density of the implants (*n* = 5 for both the groups; *p < 0.05). d) Quantitative analysis of the vessel diameter indicated that Si ions stimulated the increase of vessel diameters (*n* = 112 for both the groups). SA: hydrogels without cells; Si‐SA: Si ion–released hydrogels without cells; ACs‐SA: hydrogels with monocultured adipocytes; ACs‐Si‐SA: Si ion–released hydrogels with monocultured adipocytes; CO‐SA: hydrogels with cocultured adipocytes and HUVECs; CO‐Si‐SA: Si ion–released hydrogels with cocultured adipocytes and HUVECs.

In addition to the adipose like tissue appearance, the Si ion–releasing sodium alginate hydrogels with cocultured cells (CO‐Si‐SA) showed clear formation of blood vessel networks as demonstrated by massive blood vessels ingrowth and blood vessel branches with several millimeters in length, significantly different as compared with other groups (Figure 7a). In order to further confirm enhanced vascularization, hematoxylin and eosin (H&E) staining and cluster of differentiation 31 (CD31) immunohistochemical staining were performed, and the results are shown in **Figure** **8**. H&E staining showed that the Si ion–releasing hydrogels with cocultured cells (Figure 8a, CO‐Si‐SA) strongly stimulated the formation of big blood vessel, as demonstrated by red blood cells distribution in lumen, while no obvious angiogenesis in groups of pure hydrogels without cells were observed (Figure 8a, SA, Si‐SA). CD31 staining revealed that the CO‐Si‐SA group showed the similar vascular structure to native fatty tissue characterized by numerous microvessels connected to adipocytes (Figure 8b).^18^ Quantitation of blood vessel density and vessel diameter distribution revealed that CO‐Si‐SA group showed larger number and diameter of blood vessels than CO‐SA and ACs‐Si‐SA (Figure 8c,d). For hydrogels with cocultured cells, the majority of blood vessels formed in CO‐Si‐SA group were bigger than 25 µm in diameters, while the diameters of the vessels in CO‐SA group was smaller than 25 µm (Figure 8d). For hydrogels with monocultured adipocytes, it is clear to see that the diameter distribution of blood vessels in AC‐Si‐SA group was between 10 and 20 µm, while that in AC‐SA group was smaller than 10 µm (Figure 8d).

## 3 Discussion

The efficiency of adipogenic differentiation of BMSCs and sufficient vascularization are key issues for successful adipose tissue engineering.[[qv: 1c,3a,5,6]] Based on the multifunctional role of Si ions on the regulation of stem cell differentiation, activation of cell–cell interactions, and stimulation of blood vessel formation,[[qv: 13a,b,14]] we hypothesized that Si ions may participate in the regulation of adipogenic differentiation of BMSCs and stimulation of vascularization during adipose tissue regeneration, in particular, Si ions may affect the interactions between BMSC‐derived adipocytes and endothelial cells which contribute to angiogenesis and adipogenesis. Our results confirmed our hypothesis that Si ions indeed enhance adipogenic differentiation of HBMSCs, and significantly activated angiogenesis and adipogenesis in adipose tissue regeneration by regulating the interactions between HBMSC‐derived adipocytes and HUVECs. In vivo studies further confirmed that Si ion releasing hydrogels with the cocultured HBMSC‐derived adipocytes and HUVECs stimulate vascularized adipose tissue regeneration.

The efficient stimulation of adipogenic differentiation of HBMSCs is one of the key steps in adipose tissue engineering. Although the conventional chemical inducing based on Pittenger's method for the adipogenic differentiation of HBMSCs has been proved to be effective, the differentiation degree and efficiency is not high enough.[[qv: 2b,3a,c,5]] One of the core scientific hypothesis of the current study is that Si ions can regulate adipogenic differentiation of HBMSCs. Our results indicated that under normal growth condition without adipogenic differentiation inducer, Si ions could not stimulate adipogenic differentiation of HBMSCs. However, when HBMSCs were cultured with adipogenic differentiation medium in presence of Si ions for 21 days, the adipogenic differentiation of HBMSCs was clearly enhanced as compared with cells only cultured in adipogenic medium without Si ions. The lipid accumulation and expression of adipogenic marker genes such as PPARγ, C/EBPα, FABP4, leptin, and adiponection in cells cultured with Si ions were significantly higher than that in cells cultured without Si ions. This result suggests that Si ions are not an inducer, and rather an enhancer of adipogenic differentiation of HBMSCs. More interesting is that when HBMSCs have been induced with AM alone for 12 days, then replacing AM with normal growth medium containing Si ions and culturing further for up to 21 day, the adipogenic differentiation was further enhanced even without adipogenic medium, and the enhancement was even higher than that of the cells cultured in adipogenic medium for the same time period. This means that, once the cells are already on the way to go adipogenic differentiation, Si ions can promote continuous differentiation of HBMSCs along the adipogenic differentiation direction without adipogenic inducer and with higher efficiency than adipogenic medium. It is known that many genes are expressed in along the signaling pathway of adipogenic differentiation. It has been reported that PPARγ and C/EBPα are the key transcription factors that act as molecular switches to drive adipogenic differentiation. These molecular switches control the fate of MSCs to differentiate into adipocytes.^15^ Once the differentiation switches were activated and opened, MSCs then initiated adipogenesis by activating expression of adipogenic genes such as FABP4, leptin, and adiponection.^19^ Many studies confirmed that most growth factors or stimuli that upregulated adipogenesis ultimately exerted their effects through regulation of PPARγ and C/EBPα expression.^20^ In the present study, we found that Si ions strongly enhanced expression of PPARγ and C/EBPα in adipogenic‐induced cells. This result suggests that although Si ions are not able to induce the initiation of adipogenic differentiation, but once the differentiation has been initiated, Si ions can significantly enhance the molecular switches such as PPARγ and C/EBPα to drive the adipogenic differentiation. Therefore, the role of Si ions in the enhancement of adipogenic differentiation is possibly due to the enhanced activation of upstream molecules of adipogenic signaling pathway.

In addition to the insufficient adipogenic differentiation efficiency of HBMSCs, the lack of effective vascularization is another problem to overcome in adipose tissue engineering.[[qv: 1a,6]] Previous studies have demonstrated that the use of MSC‐derived adipocytes and endothelial cell coculture model might be an effective strategy to reconstruct engineered adipose tissue with better vascularization.[[qv: 1c]] Our previous study has confirmed that Si ions significantly stimulated angiogenesis of endothelial cells by regulating cell–cell interactions in BMSCs/endothelial cells and fibroblast/endothelial cells' coculture system.[[qv: 13a,14]] Therefore, one of our hypothesis is that Si ions may stimulate angiogenesis by regulating the interactions between HBMSC‐derived adipocytes and endothelial cells. Our results indeed demonstrated that Si ions significantly enhanced capillary‐like network formation of cocultured HUVECs by activating the cocultured adipocytes to express high level of angiogenic growth factors VEGF, which then upregulated the expression of the VEGF receptors (VEGFR2) on the cytomembrane of cocultured HUVECs. Previous studies have demonstrated that VEGF secreted by adipocytes plays an important role in enhancing angiogenesis.^21^ Lai et al. found that the blockade of VEGFR2 abolished neovascularization of HUVECs in a coculture system.^22^ In our experiments, we found that in the presence of Si ions, not only the expression of angiogenic growth factors VEGF in cocultured adipocytes was upregulated, but also the expression of VEGF receptors such as VEGFR2 in cocultured HUVECs was significantly enhanced, while cocultured adipocytes almost did not express VEGFR2. These results indicate that the stimulation of angiogenesis in HBMSC‐derived adipocyte/HUVEC coculture system by Si ions is mainly through a paracrine pathway. In addition to VEGF, another important growth factor IGF1 has been found involved in both angiogenesis and adipogenesis by interacting with its receptor IGF1R, followed by the activation of the IGF1/IGF1R signaling pathway.[[qv: 16,17,20b]] Similar to VEGF expression, our results showed that Si ions strongly upregulated the expression of IGF1 in cocultured adipocytes in the coculture system, while stimulated the expression of IGF1 in cocultured HUVECs in a lower level than cocultured adipocytes. In contrast, the IGF1 receptor (IGF1R) was not only significantly upregulated in cocultured HUVECs, but also upregulated in cocultured adipocytes by Si ions at certain degree, indicating that IGF1‐related signaling was activated by Si ions not only through paracrine, but also through autocrine pathways, which contribute to the enhancement of both angiogenesis and adipogenesis in the coculture system. In recent years, growth factors like IGF1 and VEGF have been widely used for tissue engineering applications including adipose tissue regeneration for enhanced angiogenesis and adipogenesis.[[qv: 3c,23]] However, applications of additive growth factors are still limited due to the challenge in controlled delivery and activity maintenance. Our results showed that Si ions significantly enhanced the expression of IGF1 and VEGF from cells, which indicates a new possibility to induce and utilize the intrinsic growth factors of the seeding cells for enhanced tissue reconstruction, and this approach may significantly reduce the complicity and cost of tissue engineering applications.

However, although the interactions of HBMSC‐derived adipocytes with endothelial cells can enhance angiogenesis, a rapid dedifferentiation of cocultured adipocytes has been observed, which may negatively affect adipose tissue reconstruction.^10^ In the present study, we also observed the phenomenon in the coculture experiments, in which cocultured adipocytes reduced lipid accumulation and adiponectin secretion after culturing with HUVECs. Interestingly, when Si ions were added in the coculture medium, the decrease of the lipid accumulation and adiponectin secretion was clearly reduced, and at the Si‐ion concentration of 14 µg mL^−1^, lipid accumulation and adiponectin secretion were even significantly higher than that in monocultured adipocytes. These results suggest that Si ions can inhibit the dedifferentiation of cocultured adipocytes. As an important adipogenic factor for accelerating adipose tissue regeneration, IGF1 has been found to play an important role in the regulation of adipogenesis through its interaction with its receptor IGF1R.^17^ In the present study, we found that without addition of Si ions, the IGF1 and IGF1R expression in cocultured adipocytes was clearly reduced as compared with that in monocultured adipocytes which further confirmed the dedifferentiation effects. Interestingly, when Si ions were added in coculture medium, the IGF1 and IGF1R expression in cocultured adipocytes was recovered, and was even higher than that in monocultured adipocytes at the Si‐ion concentration of 14 µg mL^−1^. These results suggest that Si ions can inhibit the dedifferentiation of cocultured adipocytes through stimulating the expression of the adipogenic factor IGF1 and its receptor IGF1R. Taken together, the activation of the interactions of the HBMSC‐derived adipocytes and HUVECs by Si ions not only enhanced angiogenesis through paracrine mechanisms, but also inhibited dedifferentiation of cocultured adipocytes, in which the activation of IGF1/IGF1R signaling pathway may play a vital role.

Considering the in vitro findings of the stimulatory effect of Si ions on adipogenesis and angiogenesis, and to further prove the applicability of the bioactive Si ions for adipose tissue engineering, we prepared a composite hydrogel with the ability to release Si ions in the bioactive concentration range. When the hydrogel was loaded with cocultured HBMSC‐derived adipocytes and HUVECs and implanted in vivo, adipose‐like tissue was formed with significantly higher vascularization than the control. These results indicated that Si ions released from biomaterials strongly stimulated vascularized adipose tissue regeneration by enhancing the interactions between cocultured adipocytes and HUVECs in vivo. Histological analysis of H&E staining and CD31 immunohistochemical staining revealed that the newly formed adipose tissue stimulated by Si ions had a structure similar to the natural fatty tissue, as demonstrated by the connection of every adipocyte to at least one capillary, which suggested possible functional adipose tissue formation.^18^ In addition, Si ions significantly promoted the formation of big blood vessels with length of several millimeters, and the self‐assembly into a network structure, which indicated the enhancement of blood vessel formation in the tissue constructs. The sufficient vascularization in turn ensured the supply with nutrients for the growth of newly formed adipose tissue.

**Figure 9:**
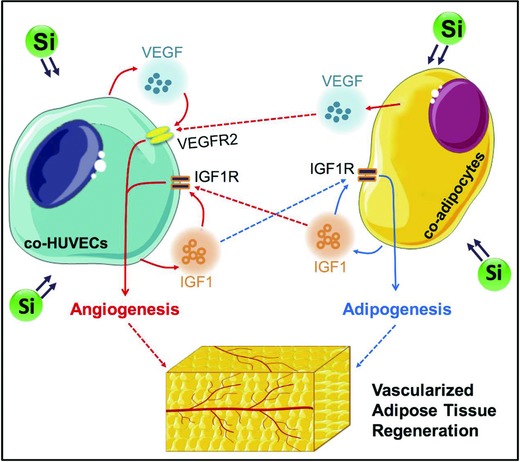
Illustration of the mechanisms of Si ions enhancing angiogenesis and adipogenesis for better vascularized adipose tissue regeneration.

Based on the in vitro and in vivo results, the possible mechanisms of the bioactive Si ions on adipose tissue reconstruction may be described as the following, as shown in **Figure** **9**. Si ions convert the chemical signal into biological signal by stimulating the interactions between HBMSC‐derived adipocytes and HUVECs, simultaneously activate adipogenesis and angiogenesis through the intracellular signal transmission, and finally enhance vascularized adipose tissue regeneration. On the one hand, Si ions strongly stimulate the expression of IGF1 mainly from cocultured adipocytes, which subsequently acted on its receptor IGF1R on the cytomembrane of co‐adipocytes, resulting in the activation of adipogenesis and inhibition of dedifferentiation, in which autocrine effects played a key role. On the other hand, Si ions remarkably enhance the expression of VEGF and IGF1 mainly from cocultured adipocytes, which subsequently act on their receptors VEGFR2 and IGF1R, respectively, on the cytomembrane of co‐HUVECs, resulting in the initiation of angiogenesis, in which paracrine effects played a key role. In summary, Si ion–enhanced adipogenesis and angiogenesis for vascularized adipose tissue regeneration may function through three possible pathways. First, Si ions enhanced adipogenesis through the enhancement of driving force of differentiation, as demonstrated by the increase of the expression of differentiation switches PPARγ and C/EBPα. Second, Si ions exerted its effects on angiogenesis of cocultured HUVECs and on the inhibition of dedifferentiation of cocultured adipocytes via stimulating the interactions of cocultured adipocytes and HUVECs, in which the promotion of the expression of growth factors IGF1 and VEGF played vital roles. Third, Si ions significantly enhanced blood vessel formation which contributed to adipogenesis and subsequently adipose tissue regeneration.

## 4 Conclusion

In this study, the bioactive effects of Si ions on adipogenic differentiation of HBMSCs were first reported. Results suggest that Si ion is an effective adipogenic differentiation enhancer of HBMSCs. Once the cells were pushed into the direction of adipogenic differentiation by adipogenic inducing medium, Si ions further pushed the cells to continue adipogenic differentiation even without the differentiation inducer, and this enhancement of adipogenic differentiation was even higher than the adipogenic medium. The possible mechanism of Si ions on the enhancement of adipogenic differentiation might be due to the activated expression of adipogenic differentiation switches such as PPARγ and C/EBPα, which were responsible for the initiation of adipogenic differentiation and further activated the expression of adipogenic genes such as FABP4, leptin, and adiponection. Furthermore, Si ions not only enhanced angiogenesis of cocultured HUVECs, but also inhibited dedifferentiation of cocultured adipocytes via affecting the interactions between HBMSC‐derived adipocytes and HUVECs. For the activation of angiogenesis, Si ions mainly stimulated the VEGF/VEGFR2 and IGF1/IGF1R signaling pathways, in which paracrine effects played a key role. For the activation of adipogenesis and inhibition of dedifferentiation, Si ions mainly stimulated the IGF1/IGF1R signaling pathway, in which autocrine effects played a key role. Finally, based on the in vitro findings of the stimulatory effect of Si ions on adipogenesis and angiogenesis, a composite hydrogel with the ability to release bioactive Si ions was designed and in vivo study further demonstrated that the bioactive hydrogel loaded with HBMSC‐derived adipocytes and HUVECs evidently stimulated the formation of vascularized adipose tissue.

## 5 Experimental Section

*Si Ion–Containing Extract Preparation and Ion Concentration Determination*: CS powders were prepared by a chemical coprecipitation method.^24^ Si ion–containing media was prepared by using CS powders based on the fact that CS releases ions gradually when soaked in cell culture medium. CS extracts were prepared according to the protocol reported in the literature,^25^ which was adapted from the standard procedure in ISO10993‐1. Briefly, CS powder was added into serum‐free DMEM (Gibco, USA) medium at a solid/liquid ratio of 200 mg mL^−1^ and incubated in a humidified incubator containing 5% CO_2_ at 37 °C for 24 h. The supernatant was collected, centrifuged (4000 rpm, 10 min), and sterilized through a filter membrane (Millipore, USA, 0.22 µm). The collected CS extracts were defined as stock solutions. Then, CS extracts were diluted with DMEM (Gibco, USA) by a series of gradient dilution at the ratios from 1 to 1/256. The Si, Ca, and P ion concentrations were measured by inductively coupled plasma atomic emission spectroscopy (ICP‐AES, Vista AX, Varian, Palo Alto, USA).

*Cell Culture*: HBMSCs were obtained from Cyagen Biosciences Inc. (China) and cultured in growth medium consisting of low glucose DMEM (Gibco, USA), 10% fetal bovine serum (FBS) (Gibco, USA), 1% penicillin–streptomycin (Gibco, USA). For adipogenic differentiation, HBMSCs were grown to postconfluence for 2 days, and then the growth medium was replaced with adipogenic differentiation A medium consisting of high glucose DMEM (Gibco, USA), 10% FBS, 1% penicillin–streptomycin, 0.5 × 10^−3^ m IBMX (Sigma‐Aldrich, USA), 10 µg mL^−1^ insulin (Sigma‐Aldrich, USA), 100 × 10^−6^ m indomethacin (Sigma‐Aldrich, USA), 1 × 10^−6^ m dexamethasone (Sigma‐Aldrich, USA) for 3 days with or without Si ions. The Si‐ion concentration used for this experiment was determined first in a preliminary screening experiment with CS extracts in the concentration range from 1.75 to 14 µg mL^−1^, and the Si concentrations of 7 and 14 µg mL^−1^ were selected due to the observed bioactive effect of these two concentrations on stimulating PPARγ expression (data not shown). Thereafter, the A medium was replaced with adipogenic differentiation B medium consisting of high glucose DMEM, 10% FBS, 1% penicillin–streptomycin, 10 µg mL^−1^ insulin for 1 day with or without Si ions. A + B represented a cycle of differentiation. 3 cycles later, cells were treated with B medium to 21 days for full development of the adipocyte phenotype. HUVECs were isolated as the previously described method.^26^ For cocultures, HBMSCs were first cultured in adipogenic differentiation medium containing Si ions for 12 days for adipogenic differentiation, and then cocultured with HUVECs containing Si ions for 3 days. HBMSCs and HUVECs used in the study were all at passage 3.

*Cell Proliferation Assay*: A Cell Counting Kit (CCK)‐8 assay was used to evaluate the effects of ionic products from CS on cell proliferation. HBMSCs and HUVECs were seeded in 96‐well plates at 1 × 10^3^ cells per well for 24 h. Then, cells were treated with media containing CS extracts for different time periods. The cell viability was measured after culturing for 3 and 7 days, respectively, using a CCK‐8 kit (Dojindo Laboratories, Japan) according to the manufacturer's instructions. Briefly, at the end of each culture time point, cell culture medium was removed, and the cells were incubated with fresh medium containing CCK‐8 reagent (10:1) for 1 h at 37 °C in an incubator. The absorbance was measured spectrophotometrically using an enzyme‐linked immunoadsorbent assay microplate reader (Epoch, BIO‐TEK, USA) at a wavelength of 450 nm.

*Actin Cytoskeleton Staining Assay*: After being cultured with different concentrations of Si ions (7 and 14 µg mL^−1^) for 7 days, cells were fixed in 4% paraformaldehyde for 30 min followed by staining with fluorescein isothiocyanate (FITC)–phalloidin (Sigma‐Aldrich, USA) for 30 min. Then, nucleus was counterstained by using 4,6‐diamidino‐2‐phenylindol (DAPI, Sigma‐Aldrich, USA) for 5 min. The morphology of the cells was visualized using a confocal laser scanning microscopy (CLSM, TCS SP8, Leica, Germany).

*Oil Red O Staining*: Differentiated adipocytes were fixed with 4% paraformaldehyde for 30 min at room temperature. Oil Red O working solution was prepared by diluting 3 mL of 0.5% Oil Red O stock solution (Sigma‐Aldrich, USA) in 2 mL of distilled water and filtered through a 0.22 µm pore‐size filter (Millipore, USA) before use. Then, cells were stained with Oil Red O working solution for 1 h and washed with distilled water to remove excess dye for photographing. For quantitative analysis, cells were destained in 100% isopropanol for 15 min and absorbance was measured at 492 nm.

*qRT‐PCR Analysis*: Total RNA was isolated using TRIzol reagent (Invitrogen, USA) according to the manufacturer's protocol. mRNA was reverse‐transcribed into complementary DNA (cDNA) using a PrimeScript RT Master Mix kit (Takara, Japan). Quantitative PCR analysis was performed by a StepOnePlus Real‐Time PCR System (Applied Biosystems, USA) using a SYBR Premix EX Taq kit (TIi RNaseH Plus, Takara, Japan) according to the instructions. Primer sequences used for qRT‐PCR analysis are shown in **Table** **2**.

**Table 2:** Primers used for qRT‐PCR

Target gene	Forward primer sequence (5′–3′)	Reverse primer sequence (5′–3′)
Glyceraldehyde 3‐phosphate dehydrogenase (GAPDH)	ACGGATTTGGTCGTATTGGGCG	CTCCTGGAAGATGGTGATGG
PPARγ	GATACACTGTCTGCAAACATATCACAA	CCACGGAGCTGATCCCAA
C/EBPα	AAGAAGTCGGTGGACAAGAACAG	TGCGCACCGCGATGT
FABP4	GCTTTGCCACCAGGAAAGTG	ATGGACGCATTCCACCACCA
Leptin	TCACACACGCAGTCAGTCTC	GAGGTTCTCCAGGTCGTTGG
Adiponectin	CCTAAGGGAGACATCGGTGA	CAATCCCACACTGAATGCTG
IGF1	ATGGGAAAAATCAGCAGTCTTC	CTACATCCTGTAGTTCTTGTTT
IGF1R	ACCCGGAGTACTTCAGCGC	CACAGAAGCTTCGTTGAGAA
VEGF	TATGCGGATCAAACCTCACCA	CACAGGGATTTTTCTTGTCTTGCT
VEGFR2	CCCAGGCTCAGCATACAA AAAGAC	CCAGTACAAGTCCCTCTGTCCC

*vWF Staining*: After coculturing for 3 days, cells were fixed for 30 min with 4% paraformaldehyde, and permeabilized with cold methanol for 5 min at room temperature, followed by the blockage with 10% goat serum for 1 h at 37 °C. Then, cells were incubated overnight with rabbit anti‐vWF antibody (Abcam, UK, 1:200) at 4 °C and then with Alexa Fluor 488 goat anti‐rabbit secondary antibody (Invitrogen, USA, 1:500) for 1 h at 37 °C. Nuclei were stained with DAPI (Sigma‐Aldrich, USA) for 5 min at room temperature. Images were then taken with confocal laser scanning microscopy (CLSM, TCS SP8, Leica, Germany).

*ELISA Analysis*: After coculture for 3 days, the culture medium was collected and centrifuged for 5 min at 10 000 rpm at 4 °C. The content of VEGF and adiponectin in the supernatant was quantified by human VEGF and adiponectin ELISA kit (Absci, USA) according to the manufacturer's protocol.

*Separation of HBMSC‐Derived Adipocytes and HUVECs Using Magnetic Beads*: To investigate the interactions between HBMSC‐derived adipocytes and HUVECs, the two cells were separated after coculturing for 3 days using magnetic beads according to the method established by Guillotin et al.^27^ The magnetic beads coupled with an antibody against CD31 (Invitrogen, USA), which is a specific protein of endothelial cells, therefore could specifically recognize and separate HUVECs from HBMSC‐derived adipocyte. The separated two cells were named co‐adipocytes and co‐HUVECs, respectively.

*Si‐SA Hydrogel Preparation*: Si‐SA hydrogel was prepared according to the previous report.[[qv: 13b]] Briefly, CS powders (1% weight) were homogeneously dispersed into a stirred 1.5% w/v alginate solution (SA) with a syringe, followed by the addition of 0.5% aspartic acid (Asp) into the alginate solution with CS. Released Ca ions from CS caused by the hydrolysis of Asp further crosslinked SA, and the gelation subsequently occurred. Pure SA hydrogel was prepared by the addition of 0.1 m CaCl_2_ solution into alginate solution as the control.

*Nude Mice Subcutaneous Implantation*: The animal experimental protocols in this study were approved by the Ethics Committee of the Shanghai Jiao Tong University School of Medicine. Before implantation, HBMSCs were first cultured in adipogenic differentiation medium containing Si ions for 21 days to achieve the adipocyte phenotype. There were 6 different groups in the implantation experiment, including SA without cells, Si‐SA without cells, SA with monocultured adipocytes, Si‐SA with monocultured adipocytes, SA with cocultured adipocytes and HUVECs, Si‐SA with cocultured adipocytes and HUVECs. For SA groups, 100 µL pure SA hydrogel encapsulating of 1 × 10^6^ cells or not was in vitro crosslinked by 0.1 m CaCl_2_. For Si‐SA groups, 100 µL pure SA hydrogel encapsulating of 1 × 10^6^ cells or not was first mixed before the addition of CS. After the gelification, hydrogels encapsulating with or without cells were implanted into subcutaneous pockets of 6 week old female nude mice which were purchased from Shanghai Laboratory Animal Center. A total of 18 mice were assigned randomly into 6 groups with 3 animals in each group. Two subcutaneous pockets on the two sides of armpits were made and only one kind of hydrogel was placed on each mouse. So for each hydrogel, there were 6 parallel samples placed on 3 animals. All mice were housed in the specific pathogen free (SPF) animal facility of the Laboratory Animal Center of the Shanghai Jiao Tong University. At 8 weeks, mice were sacrificed and implants were taken out and analyzed for blood vascular formation and adipose tissue formation.

*Histological and Immunohistochemical Analysis*: Implants were retrieved at 8 weeks and first photographed for the appearance analysis followed by the preparation for histological and immunohistochemical analysis. For adipose tissue formation analysis, the implants were fixed in 4% paraformaldehyde, embedded in optimal cutting temperature (OCT) compound, and frozen at −80 °C. Then, the samples were cut into 10 µm sections, stained with Oil Red O working solutions (Sigma‐Aldrich, USA), and counterstained with hematoxylin. For neovascularization analysis, the implants were fixed in 4% paraformaldehyde and embedded in paraffin and then cut into 5 µm sections. The sections were then subjected to deparaffinizing, rehydrating and stained with H&E (Sigma‐Aldrich, USA) as the manufacturer's instructions. For immunohistochemical staining, after deparaffinizing and rehydrating, the sections were boiled in 0.01 mol L^−1^ sodium citrate buffer solution (pH 6.0) for 10 min for antigen retrieval, and then treated with 3% H_2_O_2_ in methanol for 10 min to block the endogenous peroxidases activity. After being blocked with 5% goat serum for 1 h at room temperature, the sections were incubated at 4 °C overnight with primary antibody against CD31 (Abcam, UK, 1:300). According to the manufacturer's instructions, biotinylated secondary antibodies were then applied for 1 h, followed by the treatment with streptavidin–horseradish peroxidase conjugates. The immunoreaction was observed using 3,3*N*‐diaminobenzidine tertrahydrochloride (DAB) staining (Gene Tech, China). Finally, the samples were counterstained with hematoxylin and dehydrated and covered with coverslips. Images were observed and photographed with an optical microscope coupled with charge‐coupled device (CCD) (DMI 4000, Leica, Germany). Quantitative analysis of the adipose tissue positive stained area was performed by Image‐Pro Plus software. Quantitative analysis of the diameter of blood vessels was analyzed by Image J software.

*Statistical Analysis*: All data shown in this study were presented as means ± standard deviation (SD). Statistical analysis for determination of differences between groups was accomplished using two‐tailed analysis of variance, performed with a computer statistical program (Student's *t*‐test), and *p < 0.05 was considered statistically significant.

## Conflict of Interest

The authors declare no conflict of interest.
